# Book Notices

**Published:** 1899-02

**Authors:** 


### BOOK NOTICES.

A Practical Treatise on the Sexual Disorders of Men, by Bukk G. Carleton, M. D., Genito-Urinary Surgeon and Specialist to the Metropolitan Hospital and Polyclinic. New York: Bœricke, Runyon & Ernesty, 1898.

This very learned volume has been before the profession for the last five months, and we can cordially recommend it after an experience with it of over three months.

The pathology of the sexual organs is excellent, and the treatment, while not always homœopathic, certainly tends that way. There are details of treatment which belong to the rational therapeutics of the old school. But it has a department of therapeutics which is really homœopathic, and very good as far as it goes. We miss the repertory which should be at the end of the materia medica in this department.

In his preface the author says:

"A careful examination and analytical study of a large number of patients suffering from the various diseases of the prostate, prostatic urethra, seminal vesicles, ampullations of Henel, the testes and spermatic cords, with the numerous associated reflex symptoms, which disappear as recovery progresses, cannot but strengthen the opinion that the great majority of the so-called functional diseases of the sexual organs of man and their accompanying neuroses will be found to be reflexes from a local disease of these parts. If these lesions are recognized and properly treated, many, if not all, of the disorders of sexual function can be relieved, and a normal, moral, and sexual life re-established.

"The nervous reflexes and manifestations associated with sexual disorders have been considered in the past to be diseases *per se*, or as arising *de novo*, it being almost the unanimous opinion that the cause in general was a diseased condition of the brain, aggravated possibly by environment, or communications received through the special senses of sight, smell, and touch. The true causes were however, generally allowed to pass without attention or treatment, and untold thousands of individuals with bright minds were permitted to degenerate or become total wrecks.

"It must not, however, be inferred that the author is of the opinion that all mental and moral depravities or physical and financial failures are due to lesions within the sexual sphere, but he is distinctly of the opinion that a large percenage of these conditions arise primarily from morbid impulses which originate from irritation or local disease of these parts."

The above quotation is here given to more clearly show what is the aim and object of the author in writing this book, and what value it will have to the practitioner in his daily practice.

We trust that his notice gives the desired insight, and that he who procures a copy of it will not find it a disappointment.

THE PORCELAIN PAINTER'S SON: A Fantasy. Edited with a foreword by Samuel Arthur Jones, M. D. Philadelphia: Bœricke & Tafel, 1898. Price, cloth, $1.00; by mail, $1.05.

This tiny book is written in the style of a novel with a view of giving the reader a glimpse of the childhood days of Samuel Hahnemann.

By its style it creates a sympathy for the master not obtainable in a more pretentious narrative. It must, therefore, arouse in those who know but little of Homœopathy and nothing at all of its founder, an interest which must lead them to a serious inquiry into the merits of the new school of medicine.

To those who have consecrated their lives to the cause it sets the beloved master in a more tender light and makes his image more vivid. This seems to be its function; this seems to be the intention of the author. In his intention he has succeeded. To it is appended a lecture entitled: "Under which King, Bezonian?" This lecture was delivered in the Homœopathic Hospital at Ann Harbor, Michigan, by request of the Faculty, on the evening of April 13th.

This lecture is very sarcastic and contains many fine "cuts" at the enemies of the homœopathic school.

This little volume is a pleasant means of entertaining one's self for a half hour. It should have a place on the table in the waiting room of every physician.

THE HOMŒÓPATHIC THERAPEUTICS OF DIPHTHERIA, by C. M. Boger, M. D. Lancaster, Pa.: T. B. & H. B. Cochran, 1898.

This volume of eighty-two pages is intended by its author, as he says in the preface, to give a "bird's eye view, as it were, of the homœopathic therapeutics of diphtheria as enunciated by our best practitioners."

It consists of a preface in which is taught the true homœopathic method of selecting the remedy; a materia medica in which are given the principal indications of the principal remedies, among which we note Carbolic-acid, Amygdala-amara, Arsenicum-iodatum, Kali-permanganicum, Lac-caninum, Cyanide of Mercury, and Plumbum-iodatum, and a repertory in which the values of the remedies are given by differences in the size of the type.

The whole forms a beautifully printed little book of large 12mo dimensions, and can be carried in the pocket for convenient use at the bedside.

It is therefore a convenient and most welcome addition to the monographs that are used by the homœopathic practitioner in his great warfare against sickness and death.

The Phonendoscope and its Practical Application, with Thirty-seven illustrations; with translations of special articles by Felix Regnault, M. D., of France, and M. Anastasiades, M. D., of Greece. Translated by A. George Baker, A. M., M. D. Philadelphia: George P. Pilling & Son, 1225 Callowhill Street, 1898. Price, 50 cents; postage, 5 cents.

The Phonendoscope is a new and wonderful instrument which is now replacing the old stethoscope for examining the lungs and heart.

The amplification of the sounds in the chest obtainable through its use is incredible to those who have had no experience of its use.

Not only by it may be made out the ordinary and well-known sounds of the chest with more distinctness but even sounds of the various abdominal organs and of the fœtus in utero. It is possible, by its use, to trace out the margins of the various organs of the body and to note the outlines of the fœtus itself and determine its exact position. While thus conducting this examination by the ear, it is possible, greatly, to aid the examination by carrying on at the same time a form of percussion examination. For this purpose, the Phonendoscope being placed upon the part to be examined, say the liver, the finger is pressed lightly against the skin and then moved along so as to make a slight rubbing effect. This rubbing produces sounds not at all audible to the unaided ear but of considerable loudness when listened to with the aid of the instrument. Another way is to gently tap with the point of the finger on the part to be examined. The results so obtained being marked with a soft crayon on the naked skin, we can actually map out on the surface of the body the outlines of the organs concealed within.

The book before us describes all these processes and more. It describes the Phonendoscope and gives illustration of the instrument itself and of all the different methods.

British, Colonial, and Continental Homœopathic Medical Directory for 1899. Edited by a member of the British Homœopathic Society, and Dr. Alexander Villers, Corresponding member of the British Homœopathic Society. London: Homœopathic Publishing Company, 12 Warwick Lane, Paternoster Row, E. C.

This is a duodecimo volume of 118 pages, whose object is sufficiently explained by its title. It has been noticed in a previous edition in this journal. The present edition is much improved, especially in the continental part being much fuller and more accurate. It is a desirable book for American travelers abroad who desire homœopathic treatment. By having a copy of it in his traveling bag the tourist can always tell who is the most suitable physician to consult in any town he may be visiting.

THREE THOUSAND QUESTIONS ON MEDICAL SUBJECTS: Arranged for self-examination. With the proper references to standard works in which the correct replies will be found. Second edition, enlarged. Philadelphia: P. Blakiston's Son & Co., 1012 Walnut Street, 1899. Price, 10 cents.

This book contains, as its name imports, three thousand questions on medical subjects. The answers to these questions are not given in the book. Instead, there follows each question a compound number which, on consulting the key in the front of the book, refers for the answer to some standard book of reference, where, on a page also indicated in the number may be read the correct answer.

The standard reference books which have to be consulted are Gould's Medical Dictionary, Morris's Anatomy, and the various quiz compends which have been the specialty of Blakiston's Son & Co. for some years back.

This book is a valuable assistance for physicians who contemplate practicing in a State where they have not been heretofore registered and find it necessary to appear before a board of examiners. Such physicians can prepare themselves for the examination with confidence by following this book.

THE INTERNATIONAL MEDICAL ANNUAL for 1899 will soon be ready to place in the hands of subscribers.

This is the seventeenth year of its publication. Among the special articles will be found the following: "Practical X-Ray Work," by R. Norris Wolfenden, M. D., B. A.; "Advances in Skull Surgery," by Seneca D. Powell, M. D.; "Surgical Treatment of Paralysis," by Drs. Robert Jones, F. R. C. S., and A. H. Tubby, M. S., M. B. These articles will be freely illustrated, chiefly by reproductions from photographs. "Climatic Treatment of Consumption," by F. de Havilland Hall, M. D., F. R. C. P. An article on "Legal Decisions Affecting Medical Men," by William A. Purrington, A. B., LL. M., will be found interesting and pertinent. In response to the request of many of our subscribers there will be found an article on "The Chief Pathogenic Bacteria in the Human Subject," with descriptions of their morphology and methods of microscopical examination, by S. G. Shattock, F. R. C. S,, the Pathological Curator of the Museum of the Royal College of Surgeons, London, illustrated by a series of finely colored plates. Octavo cloth, 700 pages. Price $3.00. E. B. Treat & Co., Publishers, 241-243 West 23d Street, New York.

